# Study on the Youth Attitude Towards Food Waste in the North-West Region of Romania

**DOI:** 10.3390/foods15183208

**Published:** 2026-09-10

**Authors:** Ana-Irina Smical, Cosmin Sabo, Adrian Petrovan, Bogdan Văduva

**Affiliations:** 1 Department of Minerals Resources Engineering, Materials and Environment, Faculty of Engineering, North University Centre of Baia Mare, Technical University of Cluj-Napoca, 430083 Baia Mare, Romania; irina.smical@irmmm.utcluj.ro; 2 Department of Electrical Engineering, Electronics and Computers, Faculty of Engineering, North University Centre of Baia Mare, Technical University of Cluj-Napoca, 430083 Baia Mare, Romania; cosmin.sabo@campus.utcluj.ro (C.S.);

**Keywords:** food waste, youth attitudes, awareness, consumer behavior, Romania, sustainability, education, Theory of Planned Behavior

## Abstract

This study aims to evaluate young people’s perceptions, attitudes, and behaviors regarding food waste in the North-West region of Romania. It seeks to identify key factors influencing awareness and practices, quantify the attitude–behavior gap, and propose targeted educational and policy interventions. A cross-sectional survey was conducted between July and October 2024 using a 17-item questionnaire. Snowball sampling via social media and educational networks yielded 955 valid responses from participants aged 14–33 across six counties. Descriptive statistics, chi-square tests, Kruskal–Wallis tests with Dunn’s post hoc comparisons, Pearson and Spearman correlation analyses, and effect size calculations were performed, guided by the Theory of Planned Behavior. Respondents showed high knowledge (89.47% aggregated positive responses) and strongly positive attitudes (>90% on all items). However, a significant attitude–behavior gap emerged: while 93.51% intended to reduce food waste, only 50.05% consistently adapted portions to appetite. Family was the primary source of awareness (42.41%), whereas formal schooling contributed minimally (3.35%), despite 92.46% supporting school-based education. Knowledge correlated strongly with attitudes (r = 0.624) but only moderately with practice (r = 0.326). Results highlight the need to integrate food waste education into formal curricula and develop targeted programs bridging the gap between positive attitudes and actual behavior change among Romanian youth.

## 1. Introduction

The problem of food waste is a fundamental and urgent issue with impacts not only on the environment but also on the economy and food security [1,2,3,4]. In 2022, between 691 and 783 million people suffered from hunger, and food insecurity increased from 25.3% to 29.6%, with chronic hunger increasing from 7.9% to approximately 9.2% [5]. The United Nations Sustainable Development Goals (SDG 12.3) aim to halve per capita global food waste at the retail and consumer levels by 2030, as well as to reduce food losses along production and supply chains [6].

In 2022, the European Union generated approximately 59.2 million tons of food waste, averaging 132 kg per inhabitant [7]. Households are the primary contributors, accounting for 54% (72 kg/inhabitant). Although no specific EU legislation governs food waste management, several directives partially address this issue [8,9]. Currently, there is no clear traceability of food waste across all EU Member States, and European statistics do not accurately include specific data for Romania [7,9]. Stefan et al. [10] demonstrated that moral attitude and perceived behavioral control influence planning and shopping routines among Romanian consumers, important predictors of food waste behavior. The lack of regional studies in Romania underscores the need for localized research.

Factors contributing to higher waste among youth include newfound independence, limited budgets leading to poor planning, and experimentation with food preferences. However, Sorokowska et al. [11] demonstrated that children older than five years already develop negative attitudes toward food waste, suggesting that early education could be crucial in establishing lifelong sustainable behaviors. Studies on Generation Z’s attitudes have revealed that environmental awareness positively influences young people’s attitudes and intentions, leading to behaviors that favor reducing food waste [12]. Young people also play an essential role in shaping sustainability policies for food systems in accordance with population needs [13].

The Theory of Planned Behavior (TPB) [14] is the most frequently utilized theoretical framework in food waste studies. According to TPB, the intention to perform a certain behavior can be predicted through three main components: attitude toward the behavior (positive or negative evaluation of reducing food waste), subjective norms (perception of social pressure from family, friends, and society), and perceived behavioral control (perception of the ease or difficulty of reducing food waste). Studies have confirmed that TPB explains a substantial proportion of variance in food waste behavior (e.g., [10]).

Beyond traditional TPB components, recent research has identified additional factors: emotions (especially guilt) predict waste-reduction intentions [10,15], and habits can override positive intentions [16]. Other significant factors include meal planning, understanding food labels, portion control, and the ability to reuse leftovers [2,17].

A significant finding in the literature is the gap between attitudes and actual behavior. While awareness, education, and positive attitudes are important, they do not always guarantee action to reduce food waste [18,19]. Food waste results from repeated and complex individual choices influenced by situational factors, and the causes of food-wasting behavior are multidimensional [20]. This intention–behavior gap represents a critical challenge for interventions aimed at reducing household food waste.

Against this background, the present study makes three contributions. First, it provides a large-sample (*n* = 955), age-stratified assessment of food waste knowledge, attitudes, and practices among young people in the North-West region of Romania, which is to our knowledge the first regional-scale study of this kind in Romania since the national consumer study of Stefan et al. [10], and it is one of the few available for Central and Eastern Europe. Second, it extends TPB-based food waste research [14] by quantifying the attitude–behavior gap in this population and by separating pre-planned purchasing decisions from in-the-moment consumption decisions, a distinction with direct consequences for the design of interventions. Third, by mapping the sources through which young people actually acquire food waste awareness, it provides an empirical basis for education policy, documenting both the current dominance of the family channel and the near-absence of the school channel. Internationally, the study adds a Central and Eastern European youth sample to a literature dominated by Western European [2] and Asian [12,17] populations, enabling cross-context comparison of the attitude–behavior gap.

This study aims to evaluate young people’s perceptions, attitudes, and behaviors regarding food waste in the North-West region of Romania, identify vulnerable points, and propose solutions to increase awareness and prevent and reduce food waste generated by human consumption. Specifically, the study pursues the following objectives: (1) to assess the level of knowledge regarding food waste among youth; (2) to analyze the attitudes of young people toward reducing food waste; (3) to identify current practices related to food consumption and waste prevention; (4) to identify demographic factors associated with food waste behavior; (5) to formulate recommendations for educational policies and interventions.

## 2. Methodology

### 2.1. Research Design

This study employed a quantitative, cross-sectional, descriptive-exploratory research design to investigate young people’s attitudes, knowledge, and practices regarding food waste in the North-West region of Romania.

The study was conducted between July and October 2024 in the North-West Development Region of Romania, encompassing six counties: Maramureș, Satu Mare, Bihor, Cluj, Sălaj, and Bistrița-Năsăud. This region was selected for its demographic diversity, including both urban and rural areas with varying levels of economic development, educational infrastructure, and food-related cultural traditions. The regional focus allows for the identification of specific factors influencing food waste behavior that may differ from national or European-level patterns [10,21].

The target population consisted of young people aged 14–33 years residing in the North-West region of Romania. This age range was selected based on previous research indicating that young people and young adults (15–30 years) represent the segment most prone to food waste in the European Union [17,22,23,24]. An additional category of respondents aged 34 years and above was included to enable comparative analysis across age groups, consistent with methodological approaches used in similar studies [2,25].

### 2.2. Research Instrument

The research instrument consisted of a structured questionnaire comprising 17 items organized into five thematic sections. The questionnaire was developed based on previously validated instruments used in food waste research [10,17,26,27], with appropriate modifications to fit the Romanian context and target population. This approach is consistent with methodological standards in the field, which emphasize adapting existing validated questionnaires to ensure comparability of results while maintaining cultural relevance [28,29]. Items were selected and adapted according to three principles: (i) coverage of the study objectives and of the TPB constructs (attitudes, subjective norms, and perceived behavioral control), together with the knowledge and practice domains; (ii) demonstrated prior use in peer-reviewed food waste studies of comparable populations, from which the item formulations were drawn [10,17,26,27]; (iii) brevity and accessibility of wording, so that the questionnaire could be completed online in a few minutes by respondents as young as 14 years. The selected items were translated into Romanian, adapted linguistically and culturally to the local context, and reviewed by the research team for clarity and content adequacy before distribution.

The structured questionnaire comprised 17 items in five sections. Section A (4 items): gender, age, residence (urban/rural), education level. Section B (3 items): knowledge of food waste definition, environmental impacts, and labeling. Section C (3 items): attitudes and moral perspectives (based on TPB). Section D (3 items): behaviors including portion adjustment, use of leftovers, and meal planning. Section E (4 items): sources of awareness, perceptions of existing programs, support for school-based education. Response options included Likert scales (1–5), multiple choice, and ordinal scales.

Response options varied by item type: Likert-type scales with 5 points were used for the knowledge and attitudinal items, while the portion-adjustment practice item used a 4-point frequency scale (never, rarely, sometimes, always) scored 1–4, consistent with methodological approaches in similar studies [17,27,28]. Multiple-choice questions were used for knowledge assessment and source identification, while ordinal scales were used for frequency-related questions.

### 2.3. Sampling and Data Collection

Data collection was conducted using a snowball (chain-referral) sampling technique, a non-probability sampling method frequently employed in food waste studies due to its effectiveness in reaching geographically dispersed populations [25,29]. This sampling approach has been successfully applied in comparable research in Ireland [29], Poland [18,25], and Indonesia [12]. The initial respondents (seeds) were recruited through two complementary channels: teachers and academic staff from schools and university centers across the six counties, who distributed the questionnaire link to students in the target age range, and the research team’s institutional and personal social media networks, selected so as to cover all six counties and the full 14–33 age span. Each participant was then encouraged to forward the questionnaire link to peers. Eligibility required residence in the North-West region, and responses were screened for completeness before inclusion in the final sample.

A total of 955 valid responses were obtained. The survey was distributed online through social media platforms and educational networks, following methodological approaches used in similar studies [17,28,30]. Prior to participation, respondents were provided with clear information about the study’s purpose and assured of anonymity and confidentiality of their responses. Informed consent was obtained from all participants. For respondents under 18, parental consent was required. The online distribution method was chosen to facilitate wide geographic coverage and reach young populations highly engaged with digital platforms [12,18].

As shown in Table 1, the sample characteristics indicate a higher proportion of female respondents (66.28%), which is consistent with food-related studies where women typically have greater responsibility for cooking and shopping activities and demonstrate higher willingness to participate in surveys related to food issues [10,28]. The predominance of respondents from urban areas (61.78%) reflects the general population distribution in the region, while the educational distribution aligns with the target population of young people, with the majority being high school students (60.21%).

### 2.4. Data Analysis and Hypotheses

Descriptive statistics and data visualization were produced using Microsoft Excel. All inferential analyses (chi-square tests, Cramér’s V, Pearson and Spearman correlations, Cronbach’s α, and Kruskal–Wallis tests with Dunn’s post hoc comparisons) were performed with a scripted pipeline in Python 3.11 (pandas 3.0.2, SciPy 1.17.1, scikit-posthocs 0.14.0). The analytical approach followed established methodologies used in food waste behavior research [2,28,29].

Descriptive statistics were employed to characterize the sample and examine the distribution of responses across all variables. Frequencies and percentages were calculated for categorical variables, while measures of central tendency (mean, median) and dispersion (standard deviation) were computed for continuous and ordinal variables where appropriate. This approach is consistent with analytical methods used in comparable studies on food waste attitudes and behaviors [17,18,25].

For inferential analysis, Chi-square tests ($\chi^{2}$) were used to examine associations between categorical variables, particularly the relationships between socio-demographic characteristics (gender, age group, residence, and education level) and food waste-related knowledge, attitudes, and practices. This statistical approach has been widely applied in food waste research to identify significant differences between demographic groups [21,28,31]. Differences in the composite Knowledge and Attitude scores across the five age groups were tested with the Kruskal–Wallis *H* test, chosen because the composite scores derive from ordinal Likert-type items and the group sizes were unequal; statistically significant results were followed by Dunn’s post hoc pairwise comparisons with Bonferroni correction, with epsilon squared ($\varepsilon^{2}$) reported as the effect size. Associations among the Knowledge, Attitude, and Practice scores were quantified with Pearson correlation coefficients and, given the ordinal origin of the underlying items, verified with Spearman rank correlations as a robustness analysis.

Cross-tabulation analysis was performed to examine relationships among variables and identify patterns in responses across demographic categories. Statistical significance was set at *p* < 0.05 for all analyses, following field conventions [2,16]. Effect sizes were calculated where appropriate to assess the practical significance of observed differences.

To assess the knowledge–attitude–practice gap, a comparative analysis was conducted between self-reported attitudes and actual behavioral practices, following the methodological approach of Tarczyńska et al. [18] and Radzymińska et al. [19]. This analysis aimed to identify discrepancies between respondents’ stated intentions to reduce food waste and their reported behaviors, a phenomenon recognized as significant in food waste research [15,20].

Internal consistency was acceptable for the Attitude scale (Cronbach’s α = 0.74) and questionable for the Knowledge scale (α = 0.66), consistent with brief scales in food waste research. The reliability coefficients were consistent with those reported in similar food waste studies using brief measurement instruments [1,17].

A post-hoc power analysis was conducted to assess the adequacy of the sample size for detecting meaningful effects. For a chi-square test with df = 4 at α = 0.05, a sample size of N = 785 is required to achieve 80% power to detect small effects (w = 0.10) according to the conventions of Cohen [32]. The current sample of *n* = 955 exceeds this threshold, providing statistical power greater than 80% to detect small effects. This ensures that the study has sufficient sensitivity to identify practically meaningful associations between demographic variables and food waste-related outcomes.

### 2.5. Ethical Considerations and Limitations

Informed consent was obtained from all participants; for minors under 18, parental consent was also obtained. The study was conducted anonymously.

All data were collected anonymously, with no personally identifiable information recorded. Responses were stored securely and used exclusively for research purposes. The online survey platform ensured data encryption and protection in compliance with data protection regulations. This approach is consistent with ethical standards applied in similar research studies [17,28,30].

Several methodological limitations should be acknowledged when interpreting the results of this study. First, the use of snowball sampling may introduce selection bias, potentially limiting the generalizability of findings to the broader population of young people in the North-West region of Romania. This limitation is common in food waste research and has been noted in similar studies [10,25].

Second, a cross-sectional design captures attitudes and behaviors at a single point in time and does not allow for causal inferences or assessment of temporal changes. Longitudinal studies would be needed to establish causal relationships between variables [29]. Third, self-reported measures of food waste behavior may be subject to social desirability bias, where respondents underreport waste or overreport positive behaviors. This limitation is inherent in survey-based food waste research and has been acknowledged in methodological discussions [10,16,17].

Fourth, the predominantly female sample composition, while typical of food-related surveys, may affect the representativeness of findings across genders. Fifth, the online distribution method may have excluded potential respondents with limited internet access, particularly in rural areas. Despite these limitations, the study provides valuable insights into young people’s attitudes and practices regarding food waste in the Romanian context, contributing to the limited regional body of research on this topic.

Finally, the Knowledge scale demonstrated questionable internal consistency (α = 0.66), which may affect the precision of knowledge measurement. This limitation may be attributed to the limited number of items (k = 3) and the heterogeneous nature of the food waste knowledge domains covered. The Attitude scale showed acceptable reliability (α = 0.74). In addition, the practice construct entering the correlation analysis was measured by a single portion-adjustment item scored on a 4-point frequency scale, which cannot capture the full breadth of food waste-related behavior. Future studies should consider developing more comprehensive scales with additional items to improve measurement reliability.

## 3. Results

### 3.1. Descriptive Analysis of Knowledge

Knowledge levels were high, with an aggregated positive response rate of 89.47%. Specifically, 89.95% recognized that rational consumption reduces food waste (52% “entirely”, 37% “partially”); 92.88% knew that food waste is biodegradable and requires selective collection (60% “entirely”, 32% “partially”); and 85.66% were aware of leachate and GHG emissions from improper disposal (46% “entirely”, 39% “partially”).

Attitudes were highly positive (>90% on all three items). The belief that following specific eating habits can reduce food waste received 94.24% agreement (69% strongly agree). Future intentions to pay more attention to rational consumption were expressed by 93.51% (67% entirely). Support for school-based educational programs was expressed by 92.46% (71% very much).

**Figure 1:**
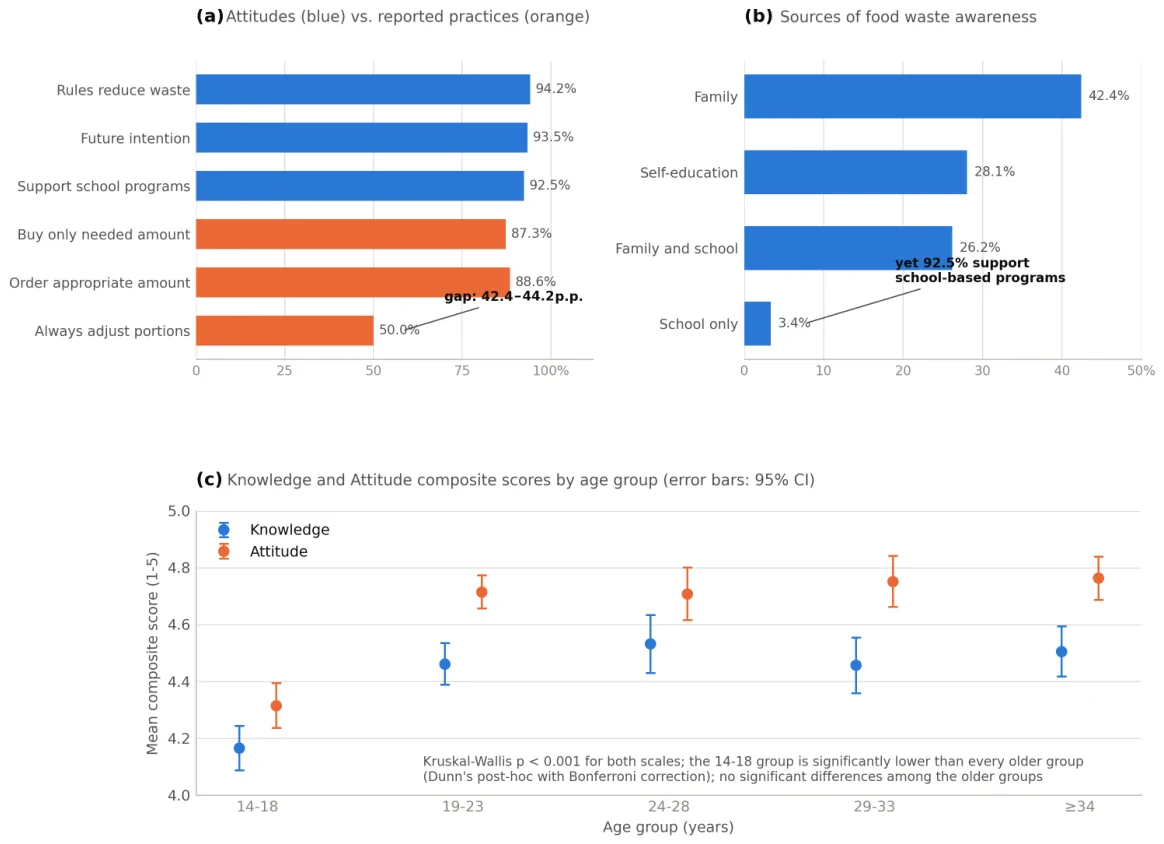
Key findings of the study (*n* = 955): (**a**) attitudes versus reported practices, with the attitude–practice gap concentrated in portion adjustment; (**b**) sources of food waste awareness, contrasting the marginal role of school with the strong support for school-based programs; (**c**) mean Knowledge and Attitude composite scores by age group (error bars: 95% confidence intervals; Kruskal–Wallis p<0.001 for both scales, with the 14–18 group significantly lower than each older group in Dunn’s post hoc comparisons).

Regarding food purchases, 87.33% reported buying only the amount needed. In restaurants, 88.59% ordered appropriate amounts. However, only 50.05% of respondents always adjusted their portions to appetite; 42.93% did so sometimes, 5.24% rarely, and 1.78% never. This represents a significant attitude–behavior gap (positive attitudes >90% vs. consistent practice ∼50%; Figure 1a).

The analysis of awareness sources revealed family as the primary contributor to food waste awareness (42.41%), followed by self-education (28.06%), a combination of family and school (26.18%), and school alone (3.35%; Figure 1b). This finding underscores the critical role of household practices and family education in shaping food-related behaviors, consistent with Stefan et al. [10] and Sorokowska et al. [11]. The marginal contribution of formal education (3.35%) represents a significant gap, particularly given that 92.46% of respondents support the implementation of school-based programs.

Regarding existing awareness programs, 43.98% of respondents perceived them as ’few,’ 17.59% as ’very few,’ and 4.50% as non-existent. The programs that do exist were attributed primarily to NGOs (27.33%), educational institutions (22.20%), and administrative authorities (14.66%), with 27.64% indicating ’other’ sources. Regarding perceptions of Romanian citizens’ awareness, only 7.64% believed Romanians are highly conscious and engaged, while 29.32% selected ’little,’ 28.90% ’partial,’ 25.76% ’very little,’ and 8.38% ’not at all,’ suggesting a perception of insufficient societal engagement with food waste issues.

### 3.2. Comparative Analysis by Demographic Variables and Hypothesis Testing

A central finding of this study is the significant gap between positive attitudes and actual behavioral practices. While attitudinal measures consistently exceeded 90% positive responses (94.24% for belief in rules, 93.51% for future intentions, 92.46% for supporting school programs), the proportion of respondents who always adjusted their portions to appetite was only 50.05%. This disparity represents a 42–44 percentage point gap between stated attitudes and consistent behavioral implementation; the gap column of Table 2 reports, for each practice item, the minimum–maximum range of the pairwise differences relative to the three attitude items (for portion adjustment, 42.4–44.2 percentage points). The gap is particularly pronounced compared to purchasing behaviors (a 5–7 percentage-point gap), suggesting that in-the-moment consumption decisions are more challenging to align with attitudes than pre-planned purchasing behaviors. This attitude–behavior gap is consistent with international findings by Tarczyńska et al. [18] and Russell et al. [15], who demonstrated that habits and emotions often override positive intentions (Table 2).

Analysis of knowledge and attitude scores across age groups revealed statistically significant differences (Kruskal–Wallis; Knowledge: H(4)=49.88, p<0.001, $\varepsilon^{2}=0.05$; Attitude: H(4)=120.27, p<0.001, $\varepsilon^{2}=0.13$; Table 3). Both scores increased with age, with the 14–18 group scoring lowest on both scales (Table 3, Figure 1c). Dunn’s post hoc comparisons with Bonferroni correction showed that the 14–18 group scored significantly lower than each older group on both Knowledge (adjusted p≤0.004) and Attitude (adjusted p<0.001), with no significant differences among the four older age groups (adjusted p≥0.55). The lower scores and higher variability among adolescents are consistent with European studies, indicating that younger individuals may have less developed attitudes and behavioral control [2,17]. Cross-tabulation of age groups with knowledge items revealed that the proportion selecting ’entirely’ increased from 41.87% in the 14–18 age group to 61.58% in the ≥34 age group, suggesting a developmental trajectory that strengthens with maturity [16].

The urban-rural comparison yielded an essentially null result: knowledge scores were identical across residence types (M = 4.36 in both groups), attitude scores differed only marginally, and the proportion purchasing only the amount needed did not differ significantly between rural (86.85%) and urban (87.63%) respondents ($\chi^{2}\left(2\right)=2.00$, p=0.367). Contrary to expectations based on [21], residence type therefore appears to play a marginal role in this Romanian sample.

Educational attainment showed a general positive association with knowledge and attitudes: high school students scored consistently lower than degree holders, and the proportion selecting ’very much’ for the belief that rules reduce waste rose from 62.43% among high school students to 81.70% among bachelor’s degree holders. This finding provides partial support for the hypothesis that higher education is associated with stronger positive attitudes, consistent with Adam et al. [28] and Secondi et al. [21].

Female respondents scored consistently higher than male respondents on both knowledge and attitudes, with the largest contrast in future intentions (72.83% of females vs. 55.74% of males selecting ’entirely’). These gender differences align with findings from Stefan et al. [10], Adam et al. [28], and Kennedy et al. [17], who observed that women tend to show higher awareness and more positive attitudes, potentially due to greater involvement in household food management.

Based on the comparative analyses, the research hypotheses were evaluated as follows (Table 4). H1 (Education influences knowledge) was partially supported: higher education levels were generally associated with higher knowledge and attitude scores, though the relationship was not perfectly linear. H2 (Urban–rural differences in practices) was not supported: contrary to expectations based on the literature, minimal differences were observed between urban and rural respondents in knowledge, attitudes, and practices, suggesting that residence type may be less influential than other demographic factors in the North-West region of Romania. H3 (Age-related differences) was strongly supported: clear age-related patterns emerged, with younger respondents (14–18 years) showing lower knowledge and attitude scores than older groups, consistent with international research [16,17].

We tested five hypotheses:

**H1.**  *Education level positively influences knowledge*

**H2.**  *Urban/rural residence influences practices*

**H3.**  *Age associated with attitude/behavior differences*

**H4.**  *Significant gap between attitudes and behavior*

**H5.**  *Family as primary source of education*

H4 (Attitude–behavior gap) was strongly supported: a substantial gap of 42–44 percentage points was identified between positive attitudes (>90%) and consistent behavioral practices (∼50%), consistent with the intention–behavior gap documented extensively by Tarczyńska et al. [18], Russell et al. [15], and Radzymińska et al. [19]. H5 (Family as primary education source) was strongly supported: family was identified as the primary source of awareness by 42.41% of respondents, far exceeding formal education (3.35%). When combined with the 26.18% who cited both family and school, family involvement reaches 68.59%, underscoring its dominant role in shaping food waste awareness, consistent with findings from Stefan et al. [10] and Sorokowska et al. [11]. These results suggest that interventions should focus on bridging the attitude–behavior gap through practical skills training, strengthening school-based programs to complement family education, and targeting younger age groups with developmentally appropriate awareness campaigns.

To assess the practical significance of the observed associations, Cramér’s V effect sizes were calculated for all chi-square tests [32]. The results revealed that while three of the four tested associations were statistically significant, the effect sizes were predominantly small: Age × Knowledge ($\chi^{2}\left(16\right)=47.06$, p<0.001, V = 0.11, small effect), Residence × Shopping behavior ($\chi^{2}\left(2\right)=2.00$, p=0.367, V = 0.05, negligible effect), Education × Attitudes ($\chi^{2}\left(16\right)=93.84$, p<0.001, V = 0.16, small effect), and Gender × Intention ($\chi^{2}\left(6\right)=50.03$, p<0.001, V = 0.16, small effect). Because several contingency cells contained few observations, all tests were re-examined after merging adjacent low-frequency response categories; the pattern of significance and the magnitude of the effect sizes remained unchanged (V = 0.14–0.18 for the significant associations). According to the guidelines of Cohen [32] (V < 0.10 negligible, 0.10–0.29 small, 0.30–0.49 medium, ≥0.50 large), these findings indicate that demographic variables explain only a small proportion of variance in food waste attitudes and behaviors.

Pearson correlation analysis was conducted to examine the relationships among the three main constructs: Knowledge, Attitudes, and Practice. The results revealed a strong positive correlation between Knowledge and Attitude scores (r = 0.624, *p* < 0.001), indicating that higher food waste awareness is associated with more positive attitudes toward waste reduction. Both Knowledge (r = 0.326, *p* < 0.001) and Attitudes (r = 0.356, *p* < 0.001) showed moderate positive correlations with actual practice behavior (measured by the portion-adjustment item). All correlations exceeded the threshold for statistical significance at emphp < 0.05 (r > 0.064 for *n* = 955). According to the guidelines of Cohen [32], the Knowledge–Attitude relationship represents a large effect, while the Knowledge–Practice and Attitude–Practice relationships represent moderate effects. These findings provide quantitative support for the attitude–behavior gap: while knowledge strongly predicts attitudes, both constructs show only moderate relationships with actual behavior. As a robustness analysis, Spearman rank correlations confirmed the same pattern (Knowledge-Attitude ρ=0.512; Knowledge–Practice ρ=0.283; Attitude–Practice ρ=0.307; all p<0.001), with a markedly stronger Knowledge–Attitude association than either association with practice, indicating that these findings are not an artifact of applying Pearson correlations to composites derived from ordinal items, a consideration particularly relevant given the modest internal consistency of the Knowledge scale (α = 0.66).

## 4. Discussion

### 4.1. Main Findings

The present study provides valuable insights into young people’s knowledge, attitudes, and practices regarding food waste in the North-West region of Romania. The findings reveal a complex picture characterized by high levels of knowledge (89.47% aggregated positive responses) and overwhelmingly positive attitudes (>90%). These results contribute to the limited body of research on food waste behavior in the Romanian context and enable meaningful comparisons with the international literature.

The high level of knowledge observed in this study is consistent with findings from similar research conducted among young populations globally. Kennedy et al. [17] reported comparable levels of knowledge among UAE residents, while Mganga et al. [30] found high awareness among Indonesian university students. Akhter et al. [1] observed similar patterns in their study of Indian university students, where the Theory of Planned Behavior successfully predicted food waste intentions. The knowledge scores in our study (M = 4.36 on a 5-point scale) are particularly comparable to those reported by Adam et al. [28] among Malaysian households, suggesting that awareness campaigns and information accessibility have achieved reasonable penetration among young populations across diverse cultural contexts.

The correlation analysis provides additional quantitative evidence for the attitude–behavior gap. While knowledge strongly predicts attitudes (r = 0.624), both constructs show only moderate relationships with actual practice (Pearson r = 0.33–0.36; Spearman ρ = 0.28–0.31), confirming that awareness and positive attitudes alone are insufficient to drive behavioral change. This pattern, observed across multiple studies [10,15], suggests that interventions targeting only knowledge and awareness may have limited effectiveness in changing actual food waste behavior. The small effect sizes observed for demographic predictors (Cramér’s V = 0.05–0.16) further indicate that individual characteristics explain only a modest proportion of variance in food waste behavior, highlighting the need to investigate contextual factors such as household composition, time constraints, and practical skill development.

Comparison with the seminal Romanian study by Stefan et al. [10] reveals both consistencies and interesting developments. Stefan et al. [10] found that planning and shopping routines were more important predictors of food waste than intentions alone among Romanian consumers. Our findings partially support this observation: while 87–88% of respondents reported appropriate purchasing and ordering behaviors, only 50% consistently adapted their portions to appetite. This suggests that pre-planned behaviors (shopping, ordering) may be more amenable to attitude alignment than in-the-moment consumption decisions, consistent with the emphasis of Stefan et al. [10] on the importance of routines and perceived behavioral control.

The attitude–behavior gap identified in this study aligns with findings from European research. Tarczyńska et al. [18] documented a similar gap among Polish students, noting that positive attitudes did not consistently translate into waste reduction behaviors. Radzymińska et al. [19] and Radzymińska and Platta [25] further confirmed this pattern, emphasizing the role of habits and automatic behaviors in overriding conscious intentions. Flanagan and Priyadarshini [29] observed similar discrepancies in their Irish study, in which environmental awareness did not always lead to behavioral change. The consistency of this gap across different European contexts suggests it represents a fundamental challenge in food waste reduction efforts rather than a culture-specific phenomenon.

Comparisons with broader European studies further contextualize our findings. Secondi et al. [21], analyzing data from 27 EU countries, found significant variation in food waste behavior across socio-demographic factors. Our finding of minimal urban–rural differences contrasts with their observations, suggesting that the Romanian context may exhibit more homogeneous patterns than the EU average. Bravi et al. [2], comparing young consumers in Italy, Spain, and the UK, found that motivations and actions to prevent food waste varied significantly across countries, underscoring the importance of context-specific research such as the present study.

Studies from Southeast Asia provide additional comparative insights. Purwanto et al. [12] found that environmental awareness significantly influenced food waste reduction intentions among Indonesian Generation Z, a pattern consistent with our findings of high environmental awareness (92.88% recognizing that food waste requires selective collection). Ismail and Azeman [27] and Adam et al. [28] reported similar attitude–behavior gaps in Malaysian contexts, reinforcing the universal nature of this challenge. The consistency of findings across diverse cultural contexts, from Romania to Indonesia to the UAE, suggests that the attitude–behavior gap in food waste may be rooted in cognitive and behavioral mechanisms that transcend cultural boundaries.

A more critical reading of these international comparisons highlights three points. First, whereas Secondi et al. [21] found significant urban–rural effects across the EU-27, our sample shows essentially none; a plausible explanation is the near-universal digital media exposure of Romanian youth a decade later, which homogenizes food-related information environments across residence types, although differences in food practices may persist in older cohorts not covered here. Second, the configuration of awareness sources distinguishes the Romanian context: where school-based food education is institutionalized, as in the Taiwanese setting studied by Kuo and Weng [31], schools act as a measurable awareness channel, whereas in our sample, the school channel is nearly absent (3.35%); the deficit is therefore institutional rather than motivational, since attitudes and support for such programs are already high. Third, the asymmetry by which pre-planned behaviors align with attitudes while in-the-moment behaviors do not replicates findings from Poland [18] and from the UK, Spain, and Italy [2], which strengthens the interpretation of this asymmetry as a robust cross-cultural feature of youth food waste behavior rather than a Romanian idiosyncrasy.

### 4.2. Theoretical Interpretation

The findings of this study can be interpreted through the lens of the Theory of Planned Behavior (TPB), which posits that behavioral intentions are determined by attitudes, subjective norms, and perceived behavioral control [14]. Our results demonstrate strong positive attitudes toward reducing food waste: 94.24% agree that following eating rules can reduce waste, and 93.51% intend to pay more attention to rational consumption. According to TPB, such positive attitudes should translate into strong behavioral intentions and, subsequently, actual behavior. However, the significant gap between attitudes and consistent behavioral practices suggests that other factors may moderate this relationship.

Perceived behavioral control emerges as a critical factor in understanding the attitude–behavior gap. Stefan et al. [10] found that perceived behavioral control (the ability to plan, shop, and prepare appropriate food quantities) was a significant predictor of food waste in Romanian households. Our finding that only 50% of respondents consistently adapt portions to their appetite, despite positive attitudes, suggests deficits in perceived or actual behavioral control. This interpretation is supported by Aydin and Yildirim [16], who demonstrated that perceived behavioral control directly influenced food waste behavior among Turkish students, and by Ghani et al. [33], who found similar patterns in Malaysian households.

The role of subjective norms, perceived social pressure to engage in or refrain from a behavior, is reflected in our findings about awareness sources. The dominance of family as the primary source of awareness (42.41% or 68.59% when including ’both family and school’) suggests that subjective norms in the household context strongly influence food waste attitudes. This finding aligns with Sorokowska et al. [11], who demonstrated that children older than five years develop negative attitudes toward food waste primarily through family socialization. However, the minimal contribution of formal education (3.35%) indicates weak institutional subjective norms, a gap that may explain why positive attitudes do not consistently translate into behaviors reinforced by social pressure outside the home.

Beyond TPB, several additional factors help explain the attitude–behavior gap. Russell et al. [15] emphasized the role of emotions and habits in food waste behavior, demonstrating that guilt and other emotional responses can both motivate and hinder waste-reduction efforts. Habits, in particular, represent automatic behavioral patterns that operate independently of conscious intentions [16]. Our finding that shopping and ordering behaviors (pre-planned) show smaller gaps than portion adjustment (in-the-moment) is consistent with habit literature: pre-planned behaviors allow for deliberate decision-making aligned with attitudes, while in-the-moment decisions are more susceptible to habitual responses.

Moral attitudes also play a significant role. Stefan et al. [10] found that moral attitudes, feelings of guilt about wasting food, predicted planning and shopping routines among Romanian consumers. Gul et al. [34] demonstrated the importance of moral and religious factors in shaping perceptions of food waste, while Chai et al. [3] explored how values influence waste-reduction intentions. The high level of support for school programs (92.46%) in our study may reflect moral awareness about the importance of addressing food waste, even when this moral awareness does not consistently guide individual behavior.

The attitude–behavior gap can also be attributed to practical barriers. Tarczyńska et al. [18] identified a lack of practical skills, time constraints, and household routines as factors preventing positive attitudes from translating into behavior. Radzymińska et al. [19] similarly noted that knowledge alone is insufficient without corresponding skills and enabling conditions. Our respondents demonstrate strong knowledge and positive attitudes but may lack practical skills, such as meal planning, portion estimation, and food storage techniques, necessary to consistently implement their intentions. This interpretation suggests that educational interventions should focus not only on awareness but also on practical skill development.

Taken together, these strands suggest a layered explanation of the attitude–behavior discrepancy observed here. At the cognitive level, portion adjustment is a repeated, low-salience decision taken under momentary appetite cues, precisely the class of behavior that habit theory predicts will escape deliberate control [15,16]. At the level of behavioral control, portion calibration requires concrete skills (estimating quantities, planning the reuse of leftovers) that, according to our awareness-source data, neither family socialization nor the school system currently teaches in a systematic way. At the normative level, family food culture in Romania, in which generous portions traditionally express hospitality and care, may work against waste-minimizing portioning; the channel that transmits anti-waste values most strongly (the family, 42.41% of awareness sources) may thus simultaneously reinforce portion norms that undermine those values. This layered account would explain why strong subjective norms against waste coexist with weak behavioral implementation, and why the gap concentrates in consumption rather than purchasing decisions.

### 4.3. Implications and Research Directions

The findings of this study have several important implications for policy and practice. First, the significant gap between support for school-based programs (92.46%) and schools’ current contribution to awareness (3.35%) clearly mandates educational policy intervention. Introducing food waste education into school curricula could address this gap, building on family education while providing systematic knowledge and skill development. Such programs should emphasize practical skills, like meal planning, portion estimation, food storage, and creative use of leftovers, rather than knowledge alone, addressing the attitude–behavior gap at its source. Similar recommendations have been made by Alattar et al. [20] based on their research among US college students and by Kuo and Weng [31] in their study of elementary school students in Taiwan.

Second, the dominance of family as a source of awareness suggests that interventions targeting households may be more effective than individual-focused campaigns. School–family partnerships could leverage both channels, with schools providing information and skills that students bring home, creating reinforcing cycles of awareness and behavior change. This approach is supported by Sorokowska et al. [11], who demonstrated that early family education shapes lasting attitudes toward food waste. Programs could also engage parents directly through workshops, digital platforms, or school-organized community events.

Third, the perception that existing awareness programs are insufficient (61.57% perceiving ’few’ or ’very few’ programs) indicates a need for increased visibility and reach. NGOs currently lead awareness efforts (27.33%), followed by educational institutions (22.20%) and administrative authorities (14.66%). Coordinated campaigns involving all stakeholders, with consistent messaging and broader reach through social media platforms favored by young people, could address this gap. The 27.64% who indicated ’other’ sources suggest that informal and community-based initiatives also play a role and could be supported and amplified.

Fourth, the age-related patterns in knowledge and attitudes suggest that interventions should be developmentally targeted. The lower scores among 14–18-year-olds indicate that adolescence represents a critical window for intervention, before attitudes and habits become more firmly established. Programs for this age group should recognize their partial dependence on family food practices while building agency and skills for independent decision-making as they transition to adulthood.

Future research directions emerging from this study include: (1) longitudinal studies tracking attitude and behavior changes over time, particularly in response to educational interventions; (2) extension to other Romanian regions to assess national patterns and regional variations; (3) qualitative research exploring the specific barriers and facilitators of translating positive attitudes into consistent behavior; (4) intervention studies testing the effectiveness of school-based programs, family-focused initiatives, and digital campaigns; (5) comparative research examining generational differences, particularly contrasting Generation Z with older generations who may have different relationships with food based on historical experiences.

## 5. Conclusions

This study investigated the knowledge, attitudes, and practices of young people aged 14–33 years regarding food waste in the North-West region of Romania. The principal findings are a high level of knowledge and strongly positive attitudes, coexisting with a substantial attitude–behavior gap concentrated in in-the-moment consumption decisions, and significant age effects in which the 14–18 group scores lowest in both knowledge and attitudes. A key finding concerns the sources of awareness: family education dominates (42.41%), while formal schooling contributes minimally (3.35%), despite 92.46% of respondents supporting the implementation of school-based programs. This discrepancy highlights a significant opportunity for educational policy intervention. The study also confirms the applicability of the Theory of Planned Behavior in the Romanian context, with positive attitudes present but perceived behavioral control and practical skills emerging as areas requiring development.

The contribution of the study is twofold. Theoretically, it extends the Theory of Planned Behavior literature to an under-studied Eastern European youth population and shows that the intention–behavior gap is concentrated in habitual, in-the-moment consumption decisions rather than in pre-planned purchasing, consistent with habit- and control-based extensions of the theory. Practically, it identifies a specific and actionable policy lever: the integration of skill-oriented food waste education into school curricula, for which the study documents both an objective need, given the near-absence of school among awareness sources, and overwhelming support among the young people themselves. The corresponding recommendations and the directions for future research are presented in Section 4.3.

In conclusion, young people in the North-West region of Romania possess the knowledge and attitudinal foundation necessary to reduce food waste, but bridging the gap to sustained behavioral change requires targeted interventions that develop practical skills, strengthen formal education, and support families as the primary educators on food. Addressing food waste among youth is not merely an environmental imperative but an investment in sustainable consumption patterns that will shape Romania’s food system for generations to come.

## Figures and Tables

**Table 1:** Socio-demographic characteristics of respondents (*n* = 955).

Variable	Category	Percentage (%)
Gender	Female	66.28
	Male	31.94
	Prefer not to answer	1.78
Age group	14–18 years	39.27
	19–23 years	20.10
	24–28 years	7.54
	29–33 years	11.83
	≥34 years	21.26
Residence	Urban	61.78
	Rural	38.22
Education level	High school	60.21
	Bachelor’s degree	23.46
	Master’s degree	14.97
	Doctoral/Post-doctoral	1.36

**Table 2:** Comparison of attitudes and behavioral practices.

Measure	Positive (%)	Gap (p.p.) ^1^
Attitude: Rules reduce waste	94.24	Reference
Attitude: Future intention	93.51	Reference
Attitude: Support school programs	92.46	Reference
Practice: Buy only needed amount	87.33	5.1–6.9
Practice: Order an appropriate amount	88.59	3.9–5.7
Practice: Always adjust portions	50.05	42.4–44.2

^1^ Each range is the minimum–maximum of the pairwise differences (in percentage points) between the given practice item and each of the three attitude items used as reference (e.g., for portion adjustment: 92.46−50.05=42.41 and 94.24−50.05=44.19). The ranges are descriptive summaries of pairwise gaps, not confidence intervals.

**Table 3:** Knowledge and attitude scores by age group.

Age Group	Knowledge M	Knowledge SD	Attitude M	Attitude SD
14–18	4.17	0.77	4.32	0.78
19–23	4.46	0.52	4.72	0.41
24–28	4.53	0.44	4.71	0.40
29–33	4.46	0.53	4.75	0.49
≥34	4.51	0.64	4.76	0.55

**Table 4:** Summary of hypothesis testing results.

Result	Support
Supported	Partial
Not supported	Weak
Supported	Strong
Supported	Strong
Supported	Strong

## Data Availability

The anonymized survey data and the complete analysis script that reproduce all statistical results reported in this article are available from the corresponding author upon reasonable request.

## Abbreviations

The following abbreviations are used in this manuscript:

TPB	Theory of Planned Behavior
SDG	Sustainable Development Goals
GHG	Greenhouse Gas
NGO	Non-Governmental Organization
EU	European Union
